# Analysis of Factors Influencing the Efficiency of Acupuncture in Tinnitus Patients

**DOI:** 10.1155/2019/1318639

**Published:** 2019-05-26

**Authors:** Tung-Yi Lin, Shih-Wei Yang, Yun-Shien Lee, Pei-Wen Wu, Chi-Kuang Young, Ting-Hua Li, Wei-Ling Chou

**Affiliations:** ^1^Department of Chinese Medicine, Chang Gung Memorial Hospital, Keelung, No. 222, Mai Chin Road, Keelung, Taiwan; ^2^School of Medicine, Chang Gung University College of Medicine, Taoyuan, Taiwan; ^3^Department of Otolaryngology-Head and Neck Surgery, Chang Gung Memorial Hospital, Keelung, No. 222, Mai Chin Road, Keelung, Taiwan; ^4^Genomic Medicine Research Core Laboratory, Chang Gung Memorial Hospital, Taoyuan, Taiwan; ^5^Department of Biotechnology, Ming Chuan University, Taoyuan, Taiwan

## Abstract

An effective acupuncture treatment must comprehend the influence of various factors, but studies in this aspect remain limited. This study aimed to identify relevant factors and search for the best practical method of acupuncture for patients with tinnitus. The study was a retrospective review of patients' data with a prospective design who had subjective idiopathic tinnitus and received acupuncture between May 2012 and August 2017. Patients' demographics, tinnitus characteristics, previous diseases, underlying diseases, oral habits, audiograms, acupuncture sessions, and acupoints were recorded and analyzed. A visual analog scale (VAS_loudness_) was used for measuring the loudness of tinnitus, and the Clinical Global Impression-Improvement scale (CGI-I) was used for assessing the suffering of patients. Good treatment responses in patients were defined as the magnitude of change from the baseline VAS_loudness_ for ≥ 30% plus CGI-I ≤ 3 points. In total, 107 patients were enrolled. Most factors were not significantly associated with the treatment effectiveness of acupuncture in tinnitus patients. Only the combination of acupoints and the number of acupuncture sessions reached statistically significant differences. Further analyzing these two factors, we confirmed that the combination of periauricular and distal acupuncture and 17 to 24 acupuncture sessions contributed to a considerably better outcome. This result would serve as a reference for clinical acupuncturists to select an appropriate acupuncture strategy in the treatment of tinnitus.

## 1. Introduction

Tinnitus is the perception of annoying sound without external auditory stimuli. Patients with tinnitus may have insomnia, anxiety, depression, inability to concentrate, and declining quality of life [1]. Severe tinnitus may also lead to serious mental illness and suicidal tendencies [2]. Tinnitus is a common symptom in adults, and it is more common in women than in men [3, 4]. Most patients have subjective tinnitus, and approximately half of the patients suffer from bilateral attacks. The frequency of tinnitus increases with age and peaks between 60 and 69 years of age [4]. A large epidemiologic study of tinnitus in America showed that approximately 10% of the Americans experienced tinnitus over the past 12 months [5]. Among 0.5% of the affected individuals, the effects of tinnitus are so severe that leading a normal life is impossible [6]. Moreover, the economic impact of tinnitus is considerable [7]. However, only approximately 20% of patients who experience tinnitus seek appropriate treatments [8].

The pathophysiology of tinnitus is unclear; its treatment is poorly effective and remains a challenge [9]. For most patients, tinnitus is a chronic disease, and its treatment aims to improve its associated symptoms and relieve its effects on the quality of life, rather than achieve an absolute cure [10]. Various therapies are recognized to treat tinnitus, including drugs, surgery, cognitive behavioral therapy, and acupuncture. However, these therapies may have a good effect on some patients but poor on others. Although cognitive behavioral (biofeedback) therapy is considered the most effective treatment for tinnitus [10], most patients with tinnitus accept simple therapies, such as taking medicine or wearing hearing aids [1]. Some researchers have pointed out that tinnitus, similar to pain, is generally a symptom for many diseases. Different tinnitus may be related to various conditions and therefore required different treatment schemes [11].

In East Asian countries, acupuncture is a usual and convenient treatment. It has been used to treat numerous diseases and symptoms, including various physiological and psychological discomforts and pain. Acupuncture therapy for tinnitus has been used for centuries, and the effect has been recommended in Chinese medical science. Acupuncture provided by well-trained physicians is relatively safe and has few side effects. Most adverse events are minor, such as bleeding, local hematoma, pain, or vegetative symptoms [12]. Several studies have demonstrated some of the positive effects of acupuncture on tinnitus [13–15]. Tinnitus may be a functional disorder related to the complex network of the body involving the central auditory and nonauditory systems [16]. It may be caused by many systemic disorders, such as autonomic nervous system disorder [17] or endocrine disorders [18, 19]. Several studies show that acupuncture can regulate the autonomic nervous system [17, 20], relieve pain [21], and regulate the endocrine system [22]. Further, acupuncture regulates neurochemicals and promotes neurogenesis and cell proliferation in the central nervous system [23]. Moreover, many patients with tinnitus suffer from a sleep disorder, and acupuncture treatment is effective in increasing the sleep quality of patients with insomnia and in improving their psychological health [24]. Therefore, the use of acupuncture therapy to treat patients with tinnitus could be promising.

In Traditional Chinese Medicine (TCM) theory, a disease occurs primarily as a consequence of the imbalance of the Yin and Yang in a human body. Each patient has a different constitution and various diagnostic patterns of TCM, representing different degrees of Yin and Yang imbalance. Acupuncture can regulate the balance by meridians and adjust imbalance in Yin and Yang to cure a disease. An effective acupuncture treatment must comprehend the patient's constitution and TCM diagnostic patterns first [9], and then the acupuncture intervention is applied according to it. Generally, the constitutions and TCM diagnostic patterns of patients are determined by considering the characteristics of the patients (such as age, sex, and body mass index), diseases (disease duration and symptoms), and other general symptoms (such as the status of defecation, sleep, and appetite). Given this, identifying relevant factors and searching for the most effective method of acupuncture are a significant concern aspect for tinnitus treatment. However, research on this aspect remains limited. For this purpose, we collected medical records data and analyzed the relationship between treatment outcomes and clinical characteristics of patients with tinnitus, including a comparison of the effectiveness between two different groups with different acupoint combinations.

## 2. Materials and Methods

### 2.1. Patient Selection

This study was a retrospective review with prospectively collected data. Patients who had tinnitus and received acupuncture treatment at the departments of Otolaryngology and Chinese Medicine of Chang Gung Memorial Hospital, Keelung, from May 2012 to August 2017, were included. All patients were diagnosed with subjective tinnitus at the clinic of otolaryngology and had undergone routine assessment. The assessment included parameters listed in the data collection section below. Patients would be excluded from this study if they (1) had received <4 sessions of acupuncture for tinnitus, (2) had discontinued therapy for >1 month during acupuncture treatment, and (3) had undergone other therapies, such as medications, surgery, and biofeedback therapy for tinnitus during the period of acupuncture therapy.

### 2.2. Acupuncture Groups

Since May 2012, the department of Chinese Medicine had set an acupuncture protocol with two different acupoint combinations for patients with tinnitus. This protocol was established in accordance with standard textbooks, previous research on acupuncture, and the experience of clinical acupuncturists [25]. One group had four periauricular acupoints (PA group), and the other group had four periauricular acupoints plus distal acupoints (PADA group). The acupoints in the PA group were TE21 (Ermen), SI19 (Tinggong), GB2 (Tinghui), and TE17 (Yifeng), and there were 11 more acupoints in the PADA group, including GV20 (Baihui), vestibulocochlear line (vertigo and auditory area), GB20 (Fengchi), TE5 (Waiguan), TE3 (Zhongzhu), KI3 (Taixi), LR3 (Taichong), SP6 (Sanyinjiao), ST36 (Zusanli), GB34 (Yanglingquan), and ST40 (Fenglong). When the patients were considering acupuncture treatment, the acupuncturist would discuss the following options with the patient. “For the treatment of tinnitus, I will do acupuncture around your affected ears. In addition to the acupoints near the ear, you can decide whether to add 11 extra acupoints to your head and body, which may or may not have additional benefit in improving your tinnitus.” Then patients were divided into PA or PADA groups according to their own choice.

### 2.3. Acupuncture Maneuver

All patients received acupuncture once or twice a week. The sterile disposable stainless needle of 0.3 mm diameter and 25 mm length was inserted into those four basic acupoints for a depth of 10–20 mm, and a needle of 0.3 mm diameter and 40 mm length was used for other distal acupoints with the approximate puncture depth of 15–30 mm. All needles were retained in each acupoint for 15–20 minutes.

### 2.4. Acupuncture Sessions

**Figure 1:**
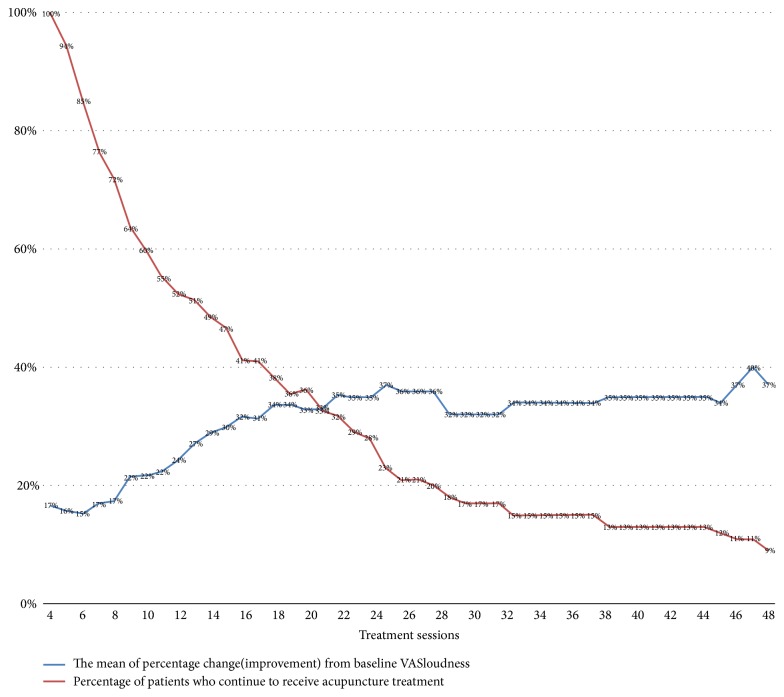
The mean of percentage change (improvement) from baseline VAS_londness_ of different acupuncture sessions and the percentage of patients (baseline = 107) who continued to receive acupuncture treatment.

Regarding the number of treatment sessions, fewer treatment sessions may be the reason for unsatisfactory results, and adequate treatment sessions are required for each tinnitus patient [26]. In our study, all patients were administered a minimum of four sessions of acupuncture treatment to ensure the possibility of successful treatment. Clinically, we usually advise patients to take acupuncture twice a week, and they will usually take 16 times in eight weeks. Besides, a previous systematic review and meta-analysis study has shown that studies with less than 17 acupuncture sessions mostly found no significant difference between the treatment group and the control group [26]. To facilitate statistical analysis, we initially divided the patients into two different acupuncture session groups (4 to 16 and more than 17 sessions) to analyze the relationship between the number of acupuncture sessions and treatment response. We noticed that the degree of improvement was insignificant after the number of acupuncture sessions reached a certain number (Figure 1). We then divided the patients into four different acupuncture sessions groups, including 4 to 8 sessions, 9 to 16 sessions, 17 to 24 sessions, and more than 25 sessions for further analysis, which could illuminate the relationship more clearly.

### 2.5. Assessment Scales

Two primary assessment scales were used for evaluating the treatment response. The visual analog scale (VAS_loudness_) was used for measuring tinnitus loudness, and the Clinical Global Impression-Improvement scale (CGI-I) was used for measuring the treatment response to suffering from tinnitus. All patients were evaluated for VAS_loudness_ and CGI-I during the treatment period. Scores were recorded on the charts for each acupuncture session.

The CGI-I is a 7-point scale that is used for measuring the improvement of the severity of illness relative to a baseline state at the beginning of the intervention. Patients rated the severity of tinnitus as (1) very much better; (2) much better; (3) minimally better; (4) no change; (5) minimally worse; (6) much worse; and (7) very much worse. The CGI-I rating scales were employed in some studies to assess a patient's subjective perception regarding the changes in the severity of tinnitus over time [27, 28].

Moreover, we considered that patients exhibited a good response to acupuncture treatment if there was a decrease in VAS_loudness_ for ≥ 30% of the baseline VAS_loudness_ plus CGI-I is ≤ 3 points. Conversely, a patient was considered to exhibit poor treatment response if the decline in VAS_loudness_ /baseline VAS_loudness_ was < 30% or CGI-I score was ≥ 4 points.

### 2.6. Data Collection

Details of the following parameters were collected and analyzed:

1. Patients' demographics: sex, age, and body mass index
2. Tinnitus history: location of tinnitus (left ear, right ear, and both ears) and duration of tinnitus. In this study, acute tinnitus is defined as tinnitus for less than three months [29]
3. Diseases of the head and neck: nasopharyngeal carcinoma, radiotherapy for head and neck cancer, chronic otitis media, and perforation of the eardrum
4. Underlying diseases that may cause tinnitus: anemia, insomnia, thyroid diseases, diabetes mellitus, dyslipidemia, and syphilis
5. Oral habits: cigarette smoking, alcohol consumption, and betel nut chewing
6. Audiograms of the patients
7. Acupuncture: the number of acupuncture sessions, acupoint groups (PA group or PADA group), VAS_loudness_, and CGI-I

### 2.7. Statistical Analysis

In this study, most parameters of patients with tinnitus were dichotomous (categorical variables). We used descriptive statistics to describe patients' characteristics, past disease history, history of tinnitus, and factors related to tinnitus. Fisher's exact test, chi-square test (BMI), and* t*-test (Audiometry) were used to evaluate the relationship between each parameter and the patient's response rate to acupuncture. Also, Fisher's exact test was used to assess the differences in patients' characteristics between the PA and PADA groups, and* t*-test was employed to analyze the differences in audiometry and BMI. For further examining the treatment response of different acupuncture combination and acupuncture sessions, we used the paired* t*-test to assess the differences before and after acupuncture treatments in the changes of VAS_loudness_ and CGI-I. Unpaired* t*-test and Fisher's exact test were used to compare the treatment responses between the PA and PADA groups. We also use the chi-square test for comparing four groups with different numbers of acupuncture sessions and calculated the mean of percentage change (improvement) from baseline VAS_loudness_ of all patients. Odds ratio (OR) and 95% confidence intervals (95% CI) were calculated using a two-tailed test of significance (*p* < 0.05) for each factor, as follows: (1) when 95% CI did not include 1.0, the resulting OR of the risk factor was statistically significant; (2) if the value of the OR was >1.0, the risk was increased; and (3) if the value was <1.0, the risk was reduced. Fisher's exact test and* t*-test were calculated using the MATLAB program (MathWorks Inc., Natick, Mass., USA).

## 3. Results

In total, 127 patients were diagnosed with tinnitus by otolaryngology specialists, and they received acupuncture treatments at the department of Chinese Medicine. Twenty patients were excluded because they did not meet the study requirements. Thus, 107 patients were enrolled and analyzed. Patients' demographic data and clinical characteristics were summarized in Table 1.

The univariate analysis related to patients receiving acupuncture treatments for tinnitus was shown in Table 2. Some factors, including sex, age, and duration of tinnitus, were not significant, while some other factors, such as the history of nasopharyngeal carcinoma, radiotherapy, eardrum perforation, chronic otitis media, thyroid dysfunction, and syphilis, were also not significant due to a small number of patients with this diseases. Only acupuncture groups (*p* = 0.026, Table 2) and the number of acupuncture sessions (*p* = 0.003, Table 2) were found to be significantly associated with the treatment outcomes in tinnitus patients.

In the evaluation the differences between PA and PADA groups, we noted that 38 (35.51%) patients were in the PA group and 69 (64.49%) in the PADA group. In the PA group, 24 patients had poor treatment responses, and 14 patients had good responses. In the PADA group, 42 patients responded well to acupuncture, and 27 patients did not. As for the patients' demographics, laboratory tests, and audiometry, there were no differences between the PA and PADA groups (Table 3). The decrease in VAS_loudness_ and CGI-I points was significant in both PA and PADA groups after the acupuncture treatment (*p* < 0.01, Table 4). Comparing the two groups, a significant decrease in the mean percentage change of baseline VAS_loudness_ (VAS_loudness_ /baseline VAS_loudness_) was found in the PADA group than in the PA group (*p* = 0.049, Table 4); also, the number of patients with the improvement in VAS_loudness_ /baseline VAS_loudness_ for ≥ 30% in the PADA group was significantly higher (*p* = 0.026, Table 4). However, there was no significant difference in the decrease of VAS_loudness_ (*p* = 0.253, Table 4) and CGI-I points after the treatment (*p* = 0.06, Table 4), and no statistical difference in the number of patients whose CGI-I was ≤ 3 points between PA and PADA groups (*p* = 0.089, Table 4).

In an analysis of the treatment sessions and efficacy of acupuncture, 17 to 24 sessions and over 25 sessions groups showed better response and were significantly better than 4 to 8 sessions group (*p* = 0.017, Table 5). Besides, we also noted that in 17 to 24 sessions group had a slightly better result but no significant difference in over 25 sessions group (Table 5). The mean of percentage change (improvement) from baseline VAS_loudness_ was gradually rising with the increase of the number of acupuncture sessions as shown in Figure 1.

Seven patients with tinnitus were successfully cured in this study, including 5 men and 2 women. Of these patients, 2 were in the PA group, and 5 were in the PADA group; 4 had suffered from acute tinnitus, 2 patients had had tinnitus for 3–6 months, and 1 patient had had tinnitus for more than 1 year (Table 6).

## 4. Discussion

Acupuncture therapy could offer some personal benefits to a specific group of patients with tinnitus [26]. But in this study, most factors were not found to influence the efficacy of acupuncture treatments, including the duration of tinnitus and age of the patients, which had been considered factors most likely influencing the effectiveness of acupuncture in tinnitus patients. Besides, factors of the history of nasopharyngeal carcinoma, radiotherapy, eardrum perforation, chronic otitis media, thyroid dysfunction, and syphilis also were not significantly relevant to the treatment outcomes, either. The small sample size may be the main reason for this result. Therefore, studies with a larger sample size are warranted for further clarification of the roles of these factors.

The distribution of the population in our study is similar to that in previous studies, in which tinnitus was slightly more prevalent in women than in men [4, 30]. In a recent cross-sectional study using the National Health Insurance Research Database in Taiwan, the ratio of men to women with tinnitus was approximately 43.1% to 56.9% [31]. It was similar to that in our study (44.9% and 55.1%). The proportions of our tinnitus patients with diabetes mellitus and stroke history were also identical, 16% as compared with 18%, and 8.3% as compared with 7.5%, respectively. Furthermore, the proportions of patients with bilateral and unilateral tinnitus in this study were 54% and 46%, respectively, which was consistent with that reported by Salvi et al. [32].

Selection and combination of appropriate acupoints according to the patients' constitution is the essential step to ensure the effectiveness of acupuncture treatment. Different acupoints exert different synergistic and antagonistic actions. In clinical practice, acupuncturists select appropriate acupoints according to the TCM theory and based on three basic principles of acupoint locations: (1) local acupoints are near the area where the symptoms occur, (2) distant acupoints along the meridian, and (3) distant acupoints based on the diagnostic patterns of TCM [33]. Generally, patients with tinnitus can be classified into 3 main TCM diagnostic patterns (also called TCM syndrome) according to the TCM theory: (1) retention of phlegm and blood stasis pattern, (2) hyperactivity of liver-yang pattern, and (3) vacuity of liver and kidney yin pattern.

The etiology of tinnitus is complex and difficult to determine. Each cause of tinnitus results in different forms of tinnitus and each of them may require a different therapeutic strategy [16]. Tinnitus is considered a symptom of a group of various diseases, and it is unlikely that a single curative treatment can achieve the goal of treatments [11, 16]. Thus, a combination of therapeutic strategies for tinnitus may be more effective than a single modality. Similarly, most patients with tinnitus had complex and multiple diagnostic patterns of TCM. According to TCM theory, selecting acupoints according to the patient's TCM diagnostic patterns would lead to a better treatment outcome. However, in clinical practice, an acupuncturist may have some problems in making a correct TCM diagnostic pattern and may choose inappropriate acupoints to treat patients with tinnitus. For this reason, most acupuncturists prefer to select more acupoints to cover the most common TCM diagnostic patterns of patients with tinnitus to obtain better results.

In the current study, there were 38 patients (35.5%) and 69 patients (64.5%) in the PA group and PADA group, respectively. Four basic acupoints near the ear (TE21, SI19, GB2, and TE17) were adopted in both groups. In the PADA group, 11 more acupoints were added according to the TCM theory and clinical research [13, 15, 26, 34, 35], including scalp acupoints (vestibulocochlear line), acupoints along the meridian, and distant acupoints. The acupoints along the meridian connecting with the ear were GB20 and GB34 (gallbladder meridian) and TE3 and TE5 (san jiao meridian). The distant acupoints were added in the PADA group to cover the most common three TCM diagnostic patterns of tinnitus. For example, KI3, LR3, and SP6 were included for the pattern of vacuity of liver and kidney yin; GV20, TE5, TE3, ST36, ST40, LR3, and SP6 were used for the retention of phlegm and blood stasis pattern; and GB20, LR3, and GB34 were used for hyperactivity of the liver-yang pattern. The treatment outcomes of the PA and PADA groups were significantly different (*p* = 0.026, OR: 2.67, CI95: 1.09–6.59, Table 2).

Patients in the PADA group may have more positive thoughts about acupuncture treatment than patients in the PA group. Positive thoughts may lead to significant differences in the characteristics of the two groups of patients, so we did a further analysis in Table 3. There were no differences between the PA and PADA groups in terms of tinnitus history, laboratory data, audiogram, and patients' clinical characteristics, which highlighted that the combination of acupoints used in the PADA group is a significant factor associated with better treatment outcomes in patients with tinnitus. Although these data could not completely rule out the influence of positive thoughts, our results could also provide acupuncturists with a reference of strategy to select appropriate acupuncture points in the clinical treatment of tinnitus. A single-blind or double-blind study in a prospective design is warranted to further elucidate the roles of positive thoughts and acupuncture in the treatment of patients with tinnitus.

A study in South Korea reported that systemic manual acupuncture showed better effects than periauricular electroacupuncture alone. The systemic manual acupuncture includes periauricular acupoints and distal acupoints in the limbs according to the meridian system theory [34]. Similarly, Rogha et al. selected acupoints for tinnitus treatment according to the diagnostic pattern of TCM, plus four basic acupoints in the periauricular region (TE17, GB2, SI19, and TE21), in which patients with tinnitus had a better response to acupuncture treatment in that study [13]. Those results are similar to the findings in the present study.

Clinically, acupuncturists often are asked by patients about the number of acupuncture sessions required for an adequate treatment course. But few studies investigated the relationship between the acupuncture sessions and the efficacy of treatments. The ideal acupuncture course needed to treat patients with tinnitus has been inconclusive so far. There is a variety of numbers of acupuncture sessions in the previous RCTs, and it ranged from 1 to 30 [9, 26, 36]. There was no apparent relationship between the numbers of acupuncture sessions and the treatment outcomes from those studies. In the present study, the patients receiving more than 17 acupuncture sessions had better therapeutic effects than those under 16 acupuncture sessions, and the VAS_loudness_ was decreased gradually with the increase of the number of acupuncture sessions (Figure 1). Furthermore, the VAS_loudness_ was reduced slowly over 25 times of acupuncture, and 17 to 24 sessions had the highest good response rate in our study (Table 5). This result demonstrates that 17 to 24 acupuncture sessions may be an effective treatment strategy for patients with tinnitus.

Clinically, most treatments of tinnitus are unsatisfied because of the complex pathophysiology of the disease, and a cure for tinnitus does not exist. Although patients with tinnitus may be beneficial from acupuncture, the treatment is still not ideal and effective. Total recovery from tinnitus is usually clinically remarkable and inexplicable. In our study, 5 patients in the PADA group and 2 patients in the PA group were cured; however, the ratio was relatively low. Of the seven patients, 4 had acute tinnitus (tinnitus < three months) and 3 had chronic tinnitus (longer than three months), including 1 patient who had had tinnitus for more than 1 year. Six of them receiving the acupuncture sessions were less than 24 times. Nevertheless, no definite conclusion can be reached because of the small sample size.

Currently, there is still no consensus on how to assess the efficacy of tinnitus treatment. We evaluated the treatment responses by using patient-reported VAS_loudness_ to determine the level of intensity of the tinnitus and patient-reported CGI-I to measure changes in the patients' subjective reports of suffering from tinnitus. In the present study, we defined a good treatment response as a decrease in VAS_loudness_ of ≥ 30% of the baseline VAS_loudness_ plus CGI-I ≤ 3 points through comprehensive analyses. Recently, several subjective questionnaires were used to evaluate the severity of tinnitus and effects of interventions, such as tinnitus handicap inventory (THI), tinnitus reaction questionnaire, tinnitus handicap questionnaire, and tinnitus questionnaire. Among these tools, THI has been proven to have excellent internal consistency, reliability, and significant correlations [37]. These questionnaires thus can be added in the future studies to evaluate the efficacy of acupuncture for patients with tinnitus.

There are some limitations to this study. First, the study was retrospective, and there was no control group so that the placebo effect could not be eliminated. Besides, the number of acupuncture sessions and the length of treatment courses varied among patients. We know that RCTs are the “gold standard” for evidence-based medicine. But in the RCTs of acupuncture, the designs of the blind and sham acupuncture of the control group are almost impossible. The acupuncture method, the combination of acupoints, the courses of acupuncture, and even results reported are also variable by different RCTs. That was the main reason of previous systematic reviews and meta-analyses reached no conclusion as to whether acupuncture has beneficial effects on the treatments of tinnitus [9, 26, 36]. The old methods, such as large databases, case series, and even case reports, still play an essential role in medical knowledge production [38]. In our opinion, it is the most concerned aspect for a clinical acupuncturist to find the most effective acupuncture method and most suitable patients. We believe the present study would provide a valuable reference for this aspect. Second, some information in patients' medical records was missing during the chart review, but the proportion was not much. Third, although patients were assessed before and after the treatment periods, the status of tinnitus was not followed up after the final acupuncture session, and it is unknown how long the effects of acupuncture could last. Forth, some critical factors, such as the depth of needles, the pitch of tinnitus, the acupuncture method with or without de-qi, and the acupuncture with or without electrical stimulation were not analyzed in this study due to the limitation of data. In future studies, those factors should be further investigated, and the tinnitus patients must be followed up for an extended period to comprehend the impact of acupuncture in patients with

## 5. Conclusions

In our study, the combination of periauricular and distal acupoints, and the patient receiving 17 to 24 acupuncture sessions, were associated with a significantly better outcome in the treatment of tinnitus patients. The results could provide a valuable reference for clinical acupuncturists to select an appropriate acupuncture strategy to treat patients with tinnitus.

## Figures and Tables

**Table 1:** Characteristics of patients who received acupuncture treatments for tinnitus (n = 107).

Characteristics	Case No. (%)
Sex (Female)	59 (55.14%)
Age (more than 65)	37 (34.58%)
BMI >25*∗*	34 (32.08%)
Affected side (Bilateral)	58 (54.21%)
Duration of tinnitus (longer than 3 months)	83 (77.57%)
History of nasopharyngeal carcinoma (Yes)	5 (4.67%)
History of radiotherapy (Yes)	8 (7.48%)
Eardrum perforation (Yes)	2 (1.87%)
Chronic otitis media (Yes)	4 (3.74%)
Cigarette smoking (Yes)	18 (16.82%)
Alcohol drinking (Yes)	11 (10.28%)
Betel nut chewing (Yes)	5 (4.67%)
Anemia† (Yes)	19 (18.81%)
Thyroid dysfunction‡ (Yes)	5 (5.49%)
Hypertriglyceridemia§ (Yes)	18 (18.0%)
Hypercholesterolemia|| (Yes)	47 (47.0%)
Syphilis¶ (Yes)	1 (1.03%)
Diabetes mellitus# (Yes)	19 (18.1%)
Insomnia (Yes)	53 (49.53%)
Cerebrovascular accident*∗∗* (Yes)	8 (7.55%)
Acupuncture treatment	
PA group	38 (35.51%)
PADA group	69 (64.49%)
Acupuncture sessions	
4 to 16	63 (58.88%)
≥ 17	44 (41.12%)
The response of treatment	
Poor	51 (47.66%)
Good	56 (52.34%)

Audiometry††	left side / right side
500 Hz, dBnHL	16.20 ± 13.04 / 16.54 ± 15.78
1000 Hz, dBnHL	20.43 ± 16.27 / 19.86 ± 19.02
2000 Hz, dBnHL	24.38 ± 19.35 / 23.75 ± 20.08
4000 Hz, dBnHL	37.93 ± 23.37 / 36.83 ± 24.36
8000 Hz, dBnHL	48.98 ± 26.45 / 47.53 ± 28.26
PB score, %	96.42 ± 8.790 / 93.96 ± 18.76

BMI: body mass index; PA: periauricular acupoints; PADA: periauricular acupoints + distal acupoints; dBnHL: decibel above normal hearing level; PB: phonetically balanced.

*∗*One piece of missing data of BMI (n = 106).

†Six pieces of missing data in the group of anemia (n = 101).

‡Sixteen pieces of missing data in the group of thyroid dysfunction (n = 91).

§Seven pieces of missing data in the group of hypertriglyceridemia (n = 100).

||Seven pieces of missing data in the group of hypercholesterolemia (n = 100).

¶Ten pieces of missing data in the group of syphilis (n = 97).

#Two pieces of missing data in the group of diabetes (n = 105).

*∗∗*One piece of missing data in the group of cerebrovascular accident (n = 106).

††Three pieces of missing data of audiometry (n = 104).

**Table 2:** Univariate analysis of the factors related to patients receiving acupuncture treatments for tinnitus.

Characteristics		Treatment outcome				
	Case No.	Poor response	Good response	p value	O.R.	95% CI
Sex (Female / Male)	59 / 48	33 /18	26 /30	0.08	2.12	0.91-4.97
Age (<65 / ≥65)	70 / 37	35 / 16	35 /21	0.546	1.31	0.55-3.17
BMI*∗* (<18.5 / 18.5 - 25 / >25)	3 / 69 / 34	1 / 35 / 15	2 / 34 / 19	0.951	0.98	0.49-1.94
Affected side (Unilateral / Bilateral)	49 / 58	25 / 26	24 / 32	0.564	1.28	0.56-2.95
Duration of tinnitus (< 3 months / ≥ 3 months)	24 / 83	9 / 42	15 / 41	0.354	0.59	0.20-1.63
History of nasopharyngeal carcinoma (No / Yes)	102 / 5	50 / 1	52 / 4	0.366	3.85	0.36-193.13
History of radiotherapy (No / Yes)	99 / 8	49 / 2	50 / 6	0.275	2.94	0.49-30.87
Ear drum perforation (No / Yes)	105 / 2	51 / 0	54 / 2	0.496	4.72	0.17- ∞
Chronic otitis media (No / Yes)	103 / 4	50 / 1	53 / 3	0.62	2.83	0.22-151.49
Cigarette smoking (No / Yes)	89 / 18	45 / 6	44 / 12	0.206	2.05	0.64-7.21
Alcohol drinking (No / Yes)	96 / 11	45 / 6	51 / 5	0.754	0.74	0.17-3.12
Betel nut chewing (No / Yes)	102 / 5	49 / 2	53 / 3	1	1.39	0.15-17.20
Anemia† (No / Yes)	82 / 19	41 / 8	41 / 11	0.615	1.38	0.45-4.37
Thyroid dysfunction‡ (No / Yes)	86 / 5	40 / 4	46 / 1	0.194	0.22	0.00-2.35
Hypertriglyceridemia§ (No / Yes)	82 / 18	37 / 10	45 / 8	0.447	0.66	0.20-2.08
Hypercholesterolemia|| (No / Yes)	53 / 47	27 / 20	26 / 27	0.429	1.4	0.59-3.33
Syphilis¶ (No / Yes)	96 / 1	46 / 0	50 / 1	1	2.76	0.02- ∞
Diabetes mellitus# (No / Yes)	86 / 19	38 / 11	48 / 8	0.317	0.58	0.18-1.76
Insomnia (No / Yes)	54 / 53	27 / 24	27 / 29	0.7	1.21	0.53-2.77
Cerebrovascular accident*∗∗* (No / Yes)	98 / 8	47 / 3	51 / 5	0.72	1.54	0.28-10.39
Acupuncture treatment				0.026	2.67	1.09-6.59
PA group	38	24	14			
PADA group	69	27	42			
Acupuncture sessions				0.003	3.62	1.59-8.24
4 to 16	63	38	25			
≥17	44	13	31			

Audiometry, left side††	104	49	55			
500 Hz, dBnHL		17.20 ± 13.37	15.28 ± 12.79	0.455	NA	NA
1000 Hz, dBnHL		21.80 ± 16.31	19.17 ± 16.27	0.412	NA	NA
2000 Hz, dBnHL		24.10 ± 17.86	24.63 ± 20.81	0.89	NA	NA
4000 Hz, dBnHL		35.80 ± 22.39	39.91 ± 24.27	0.373	NA	NA
8000 Hz, dBnHL		46.00 ± 24.72	51.79 ± 27.93	0.269	NA	NA
PB score, %		96.00 ± 8.84	96.37 ± 8.83	0.95	NA	NA
Audiometry, right side††	104	49	55			
500 Hz, dBnHL		19.50 ± 18.96	13.80 ± 11.65	0.065	NA	NA
1000 Hz, dBnHL		22.50 ± 22.43	17.41 ± 15.01	0.174	NA	NA
2000 Hz, dBnHL		26.00 ± 22.86	21.67 ± 17.07	0.274	NA	NA
4000 Hz, dBnHL		37.30 ± 27.09	36.39 ± 21.77	0.85	NA	NA
8000 Hz, dBnHL		48.00 ± 31.10	47.08 ± 25.58	0.84	NA	NA
PB score, %		91.00 ± 23.61	96.67 ± 12.38	0.127	NA	NA

O.R.: odds ratio; CI: confidence interval; NA: not available; BMI: body mass index; PA: periauricular acupoints; PADA: periauricular acupoints + distal acupoints; dBnHL: decibel above normal hearing level; PB: phonetically balanced.

*∗*One piece of missing data of BMI (n = 106).

†Six pieces of missing data in the group of anemia (n = 101).

‡Sixteen pieces of missing data in the group of thyroid dysfunction (n = 91).

§Seven pieces of missing data in the group of hypertriglyceridemia (n = 100).

||Seven pieces of missing data in the group of hypercholesterolemia (n = 100).

¶Ten pieces of missing data in the group of syphilis (n = 97).

#Two pieces of missing data in the group of diabetes (n = 105).

*∗∗*One piece of missing data in the group of cerebrovascular accident (n = 106).

††Three pieces of missing data of audiometry (n = 104).

**Table 3:** Comparison between two groups of patients who received different acupuncture treatments for tinnitus.

Characteristics	PA group	PADA group	P value
	(n = 38)	(n = 69)	
Sex (female/male ratio)	1.24	1.23	1
Age (more than 65)	9 (23.68%)	28 (40.58%)	0.092
BMI*∗*	23.28 ± 2.93	23.63 ± 3.34	0.471
Affected side (bilateral)	18 (47.378%)	40 (57.97%)	0.317
Duration of tinnitus (longer than 3 months)	29 (76.31%)	54 (78.26%)	0.813
History of nasopharyngeal carcinoma	1 (2.63%)	4 (5.80%)	0.654
History of radiotherapy	2 (5.26%)	6 (8.70%)	0.709
Ear drum perforation	1 (2.63%)	1 (1.45%)	1
Chronic otitis media	0	4 (5.80%)	0.295
Cigarette smoking	6 (15.79%)	12 (17.39%)	1
Alcohol drinking	5 (13.16%)	6 (8.70%)	0.515
Betel nut chewing	3 (7.89%)	2 (2.90%)	0.345
Anemia†	8 (21.62%)	11 (17.19%)	0.605
Thyroid dysfunction‡	0	5 (8.47%)	0.158
Hypertriglyceridemia§	4 (11.11%)	14 (21.88%)	0.278
Hypercholesterolemia||	14 (38.89%)	33 (51.56%)	0.297
Syphilis¶	1 (2.78%)	0	0.371
Diabetes mellitus#	7 (18.92%)	12 (17.65%)	1
Insomnia	20 (52.63%)	33 (47.83%)	0.689
Cerebrovascular accident*∗∗*	3 (8.11%)	5 (7.25%)	1
Acupuncture sessions(≥17)	14 (36.8%)	30 (43.5%)	0.544

Audiometry, left side††			
500 Hz, dBnHL	17.16 ± 13.57	15.67 ± 12.82	0.579
1000 Hz, dBnHL	22.84 ± 18.20	19.10 ± 15.07	0.265
2000 Hz, dBnHL	28.11 ± 21.19	22.31 ± 18.10	0.145
4000 Hz, dBnHL	42.97 ± 24.25	35.15 ± 22.56	0.102
8000 Hz, dBnHL	54.32 ± 28.65	45.98 ± 24.86	0.125
PB score, %	96.0 ± 9.61	96.66 ± 8.37	0.717
Audiometry, right side††			
500 Hz, dBnHL	16.49 ± 13.17	16.57 ± 17.15	0.980
1000 Hz, dBnHL	20.95 ± 16.74	19.25 ± 20.27	0.666
2000 Hz, dBnHL	24.86 ± 17.30	23.13 ± 21.56	0.676
4000 Hz, dBnHL	39.73 ± 23.18	35.22 ± 25.01	0.369
8000 Hz, dBnHL	51.89 ± 28.66	45.08 ± 27.95	0.250
PB score, %	95.89 ± 13.54	92.90 ± 21.18	0.438

BMI: body mass index; PA: periauricular acupoints; PADA: periauricular acupoints + distal acupoints; dBnHL: decibel above normal hearing level; PB: phonetically balanced.

*∗*One piece of missing data of BMI (n = 106).

†Six pieces of missing data in the group of anemia (n = 101).

‡Sixteen pieces of missing data in the group of thyroid dysfunction (n = 91).

§Seven pieces of missing data in the group of hypertriglyceridemia (n = 100).

||Seven pieces of missing data in the group of hypercholesterolemia (n = 100).

¶Ten pieces of missing data in the group of syphilis (n = 97).

#Two pieces of missing data in the group of diabetes (n = 105).

*∗∗*One piece of missing data in the group of cerebrovascular accident (n = 106).

††Three pieces of missing data of audiometry (n = 104).

**Table 4:** Comparison of the treatment responses between PA and PADA groups.

	PA group (n = 38)	PADA group (n = 69)	*p* value
The score change of VAS_loudness_			0.253
Before treatment	5.55 ± 2.26	5.08 ± 1.91	
After treatment	4.29 ± 2.44	3.41 ± 2.16	
Decrease in VAS_loudness_	1.26 ± 1.83	1.67 ± 1.72	
*p* value	< 0.01	< 0.01	

The percentage change of Baseline VAS_loudness_ (improvement)			0.049
Decrease in VAS_loudness_ /Baseline VAS_loudness_	0.17 ± 0.48	0.33 ± 0.32	
*p* value	< 0.01	< 0.01	

The decrease in VAS_loudness_ /Baseline VAS_loudness_			0.026
≥ 30%	14	42	
< 30%	24	27	

CGI-I			0.06
Before treatment	4	4	
After treatment	3.13 ± 1.12	2.77 ± 1.16	
*p* value	< 0.01	< 0.01	

CGI-I			0.089
CGI-I ≤ 3	21	50	
CGI-I ≥ 4	17	19	

PA: periauricular acupoints; PADA: periauricular acupoints + distal acupoints; VAS_loudness_: visual analog scale; CGI-I: Clinical Global Impression–Improvement scale.

**Table 5:** Comparison of four groups of patients who received different numbers of acupuncture sessions.

		Treatment outcome				
	Case No.	Poor response	Good response	p value	OR	95% CI
Acupuncture sessions				0.0088		
4 to 8	39	26	13		1	
9 to 16	24	12	12		2	0.71-5.66
17 to 24	19	5	14		5.6	1.65-18.95
≥ 25 (25 to 124)	25	8	17		4.25	1.45-12.42

OR: odds ratio; CI: confidence interval.

**Table 6:** Characteristics of patients with tinnitus who were successfully treated by acupuncture.

Patient	Acupuncture group	Sex	Age	Affected side	BMI	Duration (months)	Baseline VAS_loudness_	Treatment sessions	Smoking	Dyslipidemia	Insomnia	Hearing impairment
#1	PA	Female	31	Left	18.5	<3	4	48	N	Y	N	N
#2	PA	Male	53	Bilateral	25.3	3−6	5	14	Y	Y	Y	N
#3	PADA	Male	81	Right	23.4	<3	5	6	N	Y	N	Y
#4	PADA	Male	78	Bilateral	18.7	<3	3	17	Y	N	N	Y
#5	PADA	Female	49	Left	25.3	<3	5	4	N	Y	N	Y
#6	PADA	Male	71	Bilateral	NA	12−24	7	18	N	NA	Y	Y
#7	PADA	Male	26	Right	26.5	3−6	2	4	Y	Y	N	N

BMI: body mass index; VAS_loudness_: visual analog scale; PA: periauricular acupoints; PADA: periauricular acupoints + distal acupoints; NA: data not available; N: no; Y: yes.

## Data Availability

All data generated or analyzed during this study are included in this published article.
